# First report of lionfish prey from Western Florida waters as identified by DNA barcoding

**DOI:** 10.7717/peerj.9922

**Published:** 2020-09-11

**Authors:** Carlos A. Santamaria, James Locascio, Taylor M. Greenan

**Affiliations:** 1 Biology Program, Thomas University, Thomasville, GA, USA; 2 Biology Program, College of Science and Mathematics, University of South Florida Sarasota-Manatee, Sarasota, FL, USA; 3 Fisheries Habitat Ecology and Acoustics Program, Mote Marine Laboratory, Sarasota, FL, USA

**Keywords:** *Pterois volitans*, Prey, Trophic dynamics, Diet composition, Florida, COI barcoding

## Abstract

DNA barcoding was used to identify prey fragments recovered from the stomachs of lionfish harvested during the 2016 Sarasota Lionfish Derby. A total of 305 prey fragments were recovered from 50 stomachs (mean = 4.6 per stomach), of which 184 (60.3%) fragments could be identified to either species or genus when Cytochrome Oxidase I (COI) sequences were queried against the Barcode of Life Database. We identified 21 fish prey species which represented fourteen families and accounted for 95.7% of genetically identifiable prey items. The remaining prey items identified corresponded to six crustacean species. The four most common prey taxa in lionfish stomachs were *Ptereleotris calliura* (24.3%), an unidentified *Microgobius* species (20.4%), *Diplectum formosum* (14.3%), and *Apogon aurolineatus* (12.2%). The most frequently observed crustacean species, *Metapenaeopsis goodei*, was found in only three stomachs (6.1%). We also report eleven taxa as putative novel lionfish prey species, most of which are common in Florida waters. Sixteen prey items were identified as lionfish (*P. volitans*); however, it was not definitive whether these detections were due to cross contamination or cannibalization. This represents the first report of lionfish diets from Florida waters in the Eastern Gulf of Mexico based on barcoding efforts. Our results are largely congruent with previous COI barcoding based studies of lionfish diets, indicating these predators to be generalists exhibiting preferences for specific prey traits but with regional differences in their diets.

## Introduction

Native to the Indo-Pacific, lionfish (*Pterois volitans* and *P. miles*) have quickly become established in reefs and coastal habitats throughout the Western Atlantic since their introduction in the 1980s. Their settlement in this region has raised concerns of severe detrimental effects on local fauna, as lionfish have been shown to drastically reduce recruitment of native coral reef fishes (Albins & Hixon, 2008), exhibit high prey consumption rates (Green, Akins & Côté, 2011), prey on native fishes and invertebrates (Green, Akins & Côté, 2011; Côté et al., 2013; Dahl & Patterson, 2014; Harms-Tuohy, Schizas & Appeldoorn, 2016; Dahl et al., 2017) including the critically endangered wrasse *Halichoeres socialis* (Rocha et al., 2015), and drive declines in reef fish populations (Green et al., 2012; Albins, 2013, 2015). Controlled experiments exemplify these concerns, as they have shown lionfish to drive abundance declines and loss of species in Bahamian reefs at higher rates than native predators of similar sizes (Albins, 2013) and population declines of >50% in native species in experimental replicates (Ingeman, 2016). These findings underscore the importance of defining the prey preferences of lionfish in the Western Atlantic, specifically in areas where lionfish are relatively understudied.

In the Gulf of Mexico, settlement and spread of lionfish is thought to only have occurred over the past ten years. The first record of lionfish in the Gulf of Mexico (2006) is from a carcass found off the coast of Pinellas County, FL and is believed to represent a short-term individual (Schofield, 2010). In 2009 however, two lionfish were captured in the western Gulf of Mexico off the coast of Yucatan, Mexico (Aguilar-Perera & Tuz-Sulub, 2010), a record thought to be the first resulting from larval dispersal into the Gulf. Within a year of this capture, lionfish were reported along the U.S. coastline of the Gulf of Mexico. In August of 2010, lionfish were reported from waters near Cortez in Manatee County, FL (Schofield, 2010). By September of 2010, lionfish had been confirmed in the northern Gulf of Mexico, off the coasts of Florida and Alabama (Schofield, 2010).

Although widely studied in other areas of the Western Atlantic, particularly in the Caribbean, research into the predatory habits within the Gulf of Mexico remains comparatively sparse perhaps as a result of their relatively shorter invasive history. To date, documentation of lionfish prey species in the region have come from the Yucatan (Valdez-Moreno et al., 2012) and the northern Gulf of Mexico (Dahl & Patterson, 2014; Dahl et al., 2017, 2018). These studies, though varied in their sampling regime and identification approaches (i.e., molecular v. visual), indicate that lionfish in the region feed primarily on small fishes and to a lesser extent on some crustacean species with some variation associated with lionfish ontogeny. These findings are largely concordant with DNA barcoding results of lionfish prey items found in other Western Atlantic habitats, including Belize (Rocha et al., 2015), Puerto Rico (Harms-Tuohy, Schizas & Appeldoorn, 2016), and The Bahamas (Côté et al., 2013). Despite several identified taxa common among these studies, regional differences appear to exist between sites as each study has identified novel lionfish prey species. Thus, surveys of lionfish stomach contents in yet-to-be studied areas are needed to further our understanding of lionfish prey diets and preferences. This research would be valuable for understanding the local impacts of lionfish predation (Mizrahi et al., 2017) and for informing local management of lionfish (Bogdanoff et al., 2018). Towards this goal, we applied Cytochrome Oxidase I (hereafter COI) barcoding to prey fragments found in the stomachs of lionfish collected during the 2016 Sarasota Lionfish Derby where no previous studies on lionfish diets had been conducted and near the area where lionfish presence was first documented in the Gulf of Mexico.

## Materials and Methods

### Specimen collection

**Figure 1:**
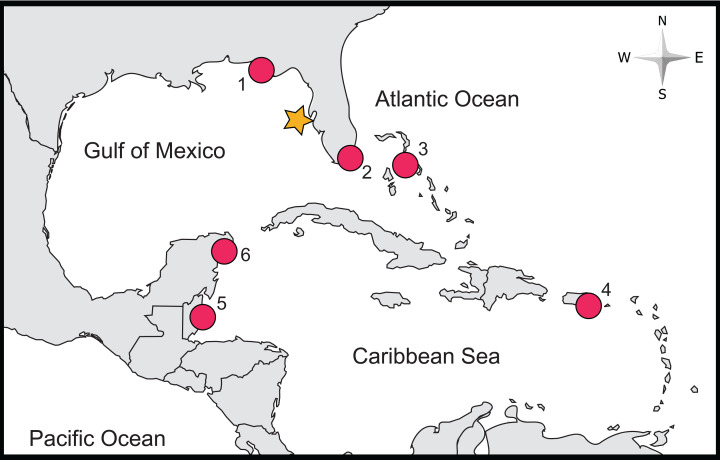
Location of study site and of previous studies of lionfish stomach contents relying on COI Barcoding. The approximate locality of this study is denoted by an orange star, while localities of previous studies are marked by red circles (labels correspond with the following studies: (1) Dahl et al. (2017), (2) Sancho et al. (2018), (3) Côté et al. (2013), (4) Harms-Tuohy, Schizas & Appeldoorn (2016), (5) Rocha et al. (2015) and (6) Valdez-Moreno et al. (2012)).

Lionfish were collected during the 2016 Sarasota Lionfish tournament in artificial habitat constructed of limestone rocks piled over the Gulfstream natural gas pipeline approximately 30 miles west of St. Petersburg, FL in 30 m water depth (Fig. 1). The limestone rubble topping sections of the pipeline creates an artificial reef in an otherwise unconsolidated sandy substrate with occasional natural hardbottom features. Lionfish were stored on ice until the removal of stomachs which was completed within 24 h of capture. Upon removal, stomachs were placed in individually labeled Whirl-Pak bags and stored at −20 °C until dissections were to be performed. Prior to dissections, the Total Length (hereafter TL) of each lionfish was recorded. Collections were carried out in accordance with all applicable local, state, and federal laws and regulations.

### Dissections and molecular work

Prey items from individual stomachs were isolated following the dissection protocol of Green, Akins & Morris (2012). During each dissection, all recognizable prey item fragments (e.g., spines, vertebrae, carapace fragments, whole fish, etc.) in each stomach were separated and inspected under a dissecting microscope. For each recognizable prey fragment, we estimated the rank of digestion (hereafter RD) per Green, Akins & Morris (2012), with prey fragments exhibiting no to minor degradation and which could be visually identified to species were assigned low RD scores (e.g., 1 and 2); highly digested items not possible to visually identify to genus or species were assigned higher scores (e.g., 4 and 5). Prey fragments were stored individually in 70% ethanol.

Total genomic DNA was extracted from each prey fragment using Zymo’s Quick-gDNA MiniPrep following standard protocol instructions. Smaller prey fragments (e.g., single spines) were used directly in DNA extractions. Fragments large enough to sub-sample (e.g., soft tissue), were dissected to obtain a small (2–5 mm) internal tissue sample as per Côté et al. (2013). Cross-contamination of samples was avoided by flame sterilizing tools after rinsing in 70% ethanol and as per Dahl et al. (2017). A 313-bp section of the COI gene was then amplified using the mlCOIintF forward (5′-GGWACWGGWTGAACWGTWTAYCCYCC) and jgHCO2198 reverse (5′-TAIACYTCIGGRTGICCRAARAAYCA) primers designed for the study of prey gut items by Leray et al. (2013) following the reaction conditions used by Harms-Tuohy, Schizas & Appeldoorn (2016). PCR mixtures consisted of 10 µL of QIAGEN Multiplex PCR mix, 7.8 µL of PCR-grade water, 0.6 µL of 10 µm of each primer, and 1 µL of genomic DNA with PCR reactions consisting of 16 initial cycles of denaturation for 10s at 95 °C, annealing for 30s at 62 °C, and extension for 60s at 72 °C, followed by 25 cycles with identical denaturation and extension steps but annealing at 46 °C, and lastly a final extension cycle of 6 min at 72 °C. For a subset of samples, we amplified a larger (658-bp) section of the COI gene using the LCO-1490 forward (5′-GGTCAACAAATCATAAAGATATTGG) and HCO-2198 reverse (5′-TAAACTTCAGGGTGACCAAAAAATCA) primers designed by Folmer et al. (1994) using PCR mixtures were as described above, with PCR reactions consisting of 35 cycles of denaturation for one minute at 95 °C, annealing for one minute at 40 °C, and extension for one and a half minute at 72 °C, with a final extension cycle of seven minutes at 72 °C. Positive amplicons were identified using 1% agarose gels stained using SYBR Safe (Life Technologies Corporation) prior to sequencing at the University of Arizona Genetics Core.

Sequences were visually inspected, assembled, and edited (i.e., primers removed) using Geneious v8.0.5. Short sequences (final length <200-bp), those displaying signs of cross-amplification or evidence suggestive of pseudo-genes (e.g., early stop codons) were not included in our analyses.

The Barcode of Life Database (BOLD) (Ratnasingham & Hebert, 2007) was used to establish the identity of each successfully sequenced prey fragment. BOLD searches were carried out using the “All Barcode Records on BOLD” option during September of 2018 with taxonomic identifications based on the following criteria. Queries which produced matches of >98% similarity were considered confident species-level identifications. Those which resulted in similarity scores of 90–98% scores were considered confident genus-level identifications provided that: (a) the queried sequence clustered within a monophyletic clade composed solely of members of a single genus in the Tree Based identifications provided by BOLD, and (b) no other genera produced similar match scores. Similar conditions were used to identify sequences producing matches of 80–90% to families. These categories were chosen because they largely correspond with the average barcoding gaps reported for teleost fish in various regions (Zhang & Hanner, 2012; Knebelsberger et al., 2014; Chang et al., 2017). Sequences not confidently identified to species but resulting in a confident genus or family level identification were combined into groups of highly similar sequences (>98% similarity) using the assembly tool of Geneious R8 to ensure that no multiple unidentified species were present in our dataset. These groups are considered as operating taxonomic units for the rest of the study (OTUs). All taxonomic identifications were checked against GenBank’s BLASTn results; however, we observed no discrepancies.

The median, and mode RD were calculated for all prey fragments, those successfully amplified and sequenced, and those which failed to amplify. We also estimated the abundance of each prey species as the number of lionfish stomachs which contained a given prey species and the frequency of occurrence of each prey species as the percentage of stomachs which contained a given prey species.

## Results

A total of 70 stomachs were dissected from lionfish ranging in size from 140 to 400 mm total length (TL; mean_TL_= 271.71 mm (SD = 69.86); median = 270 mm; mode = 269 mm; Interquartile Range: 221.5−332.5 mm). Twenty of the stomachs did not contain any observable prey fragments (28.6%). These individuals did not statistically differ in size from those which contained preys fragments (*t*(68) = −1.48987; *p*-value = 0.1410). In the remaining fifty stomachs (71.4%), we recovered 305 prey fragments (per stomach mean = 6.1, median = 4, mode =1). Of these, 184 prey fragments from 49 lionfish stomachs produced sequences meeting the minimum quality criteria (e.g., short sequences, evidence of cross-amplification). The remaining 121 fragments either failed to amplify despite several PCR attempts or produced sequences of low quality. This represents a 60.3% success rate of amplification. Most fragments were highly digested (RD: median = 4.5; mode = 5), preventing effective visual identification. Fragments that failed to amplify and/or produced low quality sequences were slightly more digested (median RD = 4.5; mode = 5) than those successfully amplified (median RD = 4; mode = 4); however, this difference was not significant (*t*(303) = 0.3253, *p* = 0.7452).

BOLD-based identifications produced species or genus level-matches for all but three sequences; 67.4% of all sequences were identified to species and 31.5% to a genus. Of these, sixteen fragments were identified as lionfish (*P. volitans*). Although cannibalism is known to occur in lionfish in the Western Atlantic (Valdez-Moreno et al., 2012; Dahl et al., 2018), we were unable to determine whether sequences identified as *P. volitans* were the result of cannibalism or cross-contamination. We thus removed these sequences from further analyses. Remaining fragments represented 21 fish species and six crustacean species. Fish prey species accounted for most obtained sequences (95.7%) and were present in the majority of lionfish stomachs (*N*_stomachs_ = 41). The most commonly observed fish species were *Ptereleotris calliura* (Blue goby; *N*_stomachs_ = 12; 24.3% of stomachs containing prey fragments), an unidentified species of *Microgobius* (*N*_stomachs_ = 10; 20.4%), *Diplectrum formosum* (Sand perch; *N*_stomachs_ = 7; 14.3%), *Apogon aurolineatus* (Bridle cardinalfish; *N*_stomachs_ = 6; 12.2%), *Heamulon aurolineatum* (Tomtate grunt; *N*_stomachs_ = 5; 10.2%). At the family level, the 21 fish species represented fourteen families. The most frequently observed fish families were Microdesmidae (Dartfishes; *N*_stomachs_ = 12; 24.5%), Apogonidae (Cardinalfishes; *N*_stomachs_ = 11; 22.4%), Gobiidae (True gobies; *N*_stomachs_ = 11; 22.4%), and Serranidae (Seabasses and Groupers; *N*_stomachs_ = 8; 16.3%). Crustacean species belonged to six crustacean families all in the Class Malacostraca. With the exception of *Metapenaeopsis goodei* (Caribbean velvet shrimp; *N*_stomachs_ = 3; 6.1%), all crustacean species were observed in a single stomach. Details of the occurrence of each species and family found in lionfish stomachs are presented in Table 1.

**Table 1:** List of prey species identified from lionfish (*Pterois* spp.) stomachs collected in waters off St. Petersburg, FL. Numbers outside parentheses indicate the number of stomachs in which each organism was found. Asterisks denote putative novel prey species. Bold terms indicate higher taxonomic levels.

Phylum Chordata Class Actinopterygii		Phylum Arthropoda Class Malacostraca	
**Family Apogonidae**	**11 (22.4%)**	**Family Alpheidae**	**1 (2.0%)**
*Apogon aurolineatus*	6 (12.2%)	Unidentified species	1 (2.0%)
*Apogon maculatus*	3 (6.1%)	**Family Gammaridae**	**1 (2.0%)**
*Phaeoptyx* sp.*	3 (6.1%)	Unidentified species	1 (2.0%)
**Family Blennidae**	**1 (2.0%)**	**Family Gonodactylidae**	**1 (2.0%)**
*Parablennius marmoreus*	1 (2.0%)	Neogonodactylus bredini*	1 (2.0%)
**Family Callionymidae**	**1 (2.0%)**	**Family Palaemonidae**	**1 ( 2.0%)**
Diplogrammus pauciradiatus*	1 (2.0%)	Unidentified species	1 (2.0%)
**Family Carangidae**	**3 (6.1%)**	**Family Penaeidae**	**3 (6.1%)**
*Decapterus punctatus*	3 (6.1%)	Metapenaeopsis goodei*	3 (6.1%)
Family Chaenopsidae	3 (6.1%)	**Family Portunidae**	1 (2.0%)
*Chaenopsis* sp.*	3 (6.1%)	Achelous ordwayi*	1 (2.0%)
**Family Gobiidae**	**11 (22.4%)**		
*Coryphopterus* sp.*	2 (4.1%)		
*Microgobius* sp.*	10 (20.4%)		
**Family Haemulidae**	**5 (10.2%)**		
*Haemulon aurolineatum*	5 (10.2%)		
**Family Labridae**	**2 (4.0%)**		
*Halichoeres* sp.*	1 (2.0%)		
*Halichoeres bivittatus*	1 (2.0%)		
**Family Microdesmidae**	**12 (24.5%)**		
*Ptereleotris calliura*	12 (24.5%)		
**Family Monacanthidae**	**3 (6.1%)**		
*Monacanthus ciliatus*	3 (6.1%)		
**Family Pomacentridae**	**5 (10.2%)**		
*Chromis scotti*	3 (6.1%)		
*Stegastes variabilis*	3 (6.1%)		
**Family Scaridae**	**1 (2.0%)**		
Sparisoma atomarium*	1 (2.0%)		
**Family Serranidae**	**8 (16.3%)**		
*Diplectrum formosum*	7 (14.3%)		
Hypoplectrus floridae*	1 (2.0%)		
**Family Synodontidae**	**8 (16.3%)**		
*Synodus intermedius*	7 (14.3%)		
Synodus saurus*	1 (2.0%)		

## Discussion

Previous studies have shown lionfish in the Western Atlantic feed primarily on fish species and that a large degree of overlap in the taxonomic composition of prey exists across the region (Valdez-Moreno et al., 2012; Côté et al., 2013; Rocha et al., 2015; Harms-Tuohy, Schizas & Appeldoorn, 2016; Dahl et al., 2017, 2018; Sancho et al., 2018). By applying molecular approaches to identify prey fragments recovered from lionfish stomachs collected in the Gulf of Mexico off the South Florida coastline we report patterns broadly consistent with previous findings. Our results also demonstrate that adult lionfish in the study region preyed primarily on fish rather than invertebrates, as reflected in both the frequency in which each prey type was observed (i.e., 41 stomachs contained at least one fish prey item, while six stomachs had at least one invertebrate prey item) and the taxonomic richness of each prey type (e.g., 21 fish prey species compared to six crustacean species). Most prey species identified in this study were previously reported as lionfish prey in the Western Atlantic (Peake et al., 2018). The three higher taxa found in >20% of surveyed lionfish stomachs in this study were prevalent in lionfish stomachs in other areas of the Western Atlantic: with Apogonidae being prevalent in Puerto Rico (Harms-Tuohy, Schizas & Appeldoorn, 2016) and the northern Gulf of Mexico (Dahl et al., 2017), Gobiidae in Belize (Rocha et al., 2015), the Yucatan peninsula (Valdez-Moreno et al., 2012), the Bahamas (Côté et al., 2013), Biscayne National Park (Sancho et al., 2018), and the northern Gulf of Mexico (Dahl et al., 2017), and Microdesmidae in the Northern Gulf of Mexico (Dahl et al., 2017).

Such congruence in the taxonomic composition of lionfish prey amongst studies underscores that while generalists, these predators exhibit preferences for specific prey traits. Most fish prey species identified in this study share at least one of the following characteristics: they are reef-associated or demersal species with average sizes of <15 cm and are mostly active at night or during crepuscular times (Matheson et al., 2017). This is best exemplified by the most frequently observed prey taxa in this study, *Ptereleotris calliura*. This species was observed in ~25% of lionfish stomachs and is a small burrowing fish which does not exceed 12.5 cm in length (Robins & Ray, 1986). Similarly, *Apogon* species are small, nocturnal, reef associated fish (Hoese & Moore, 1998) and *Microgobius* are small reef-associated fish that may display burrowing behaviors (Birdsong, 1981). *Diplectrum formosum* while growing to relatively larger adult sizes than the above prey species, is a demersal species commonly associated with sandy and coarse gravel bottoms (Hoese & Moore, 1998). Lastly, *Haemulon aurolineatum* is a nocturnal reef-associated species that are <11 cm long in the juvenile stage (Collette & Talbot, 1972; Manooch & Barans, 1982). Such apparent preferences are in concordance with the findings of Green & Côté (2014), who using a trait-based model, identified nocturnal, small, shallow-bodied, solitary fishes found resting on or just above reefs as the most vulnerable to lionfish predation. Thus, our findings support the idea that lionfish, while generalists, exhibit a preference for small, nocturnal, reef associated fish prey in the Western Atlantic (see Peake et al. (2018) and references therein).

It is important to note that despite the overlap amongst results and past studies, we report patterns that suggest regional differences in lionfish diets may exist. We identified eleven putative novel lionfish prey taxa, nine species not identified in previous barcoding studies of lionfish stomach contents in the Western Atlantic [*Diplogrammus pauciradiatus* (Spotted dragonet), *Halichoeres caudalis* (Painted wrasse), *Hypoplectrus floridae* (Florida Hamlet), *Phaeoptyx xenus* (Sponge cardinalfish), *Sparisoma atomarium* (Greenblotch parrotfish), *Synodus saurus* (Atlantic lizardfish); Crustacean: *Achelous ordwayi* (Redhair swimming crab), *Metapenaeopsis goodei* (Caribbean velvet shrimp), *Neogonodactylus bredini* (Caribbean rock mantis shrimp)] and two unidentified species from genera known to be lionfish prey (*Chaenopsis* sp. and *Microgobius* sp.). Based on observations reported in the Smithsonian Tropical Research Institute Online Information System (Robertson & Van Tassell, 2019) as well as the Global Biodiversity Information Facility databases (www.gbif.org), most of these novel prey taxa appear to be more prevalent in Florida waters than in those previously surveyed by barcoding studies. These findings indicate at least a partial effect of differences in regional assemblages in lionfish prey composition. Indeed, although comparisons across barcoding studies of lionfish in the Western Atlantic are hampered by the application of different methodologies and the reporting of different frequency statistics, both prey composition and frequencies observed in our study appear to be the most similar to those reported by Dahl et al. (2017) from nearby Northern Gulf of Mexico waters (see Tables S1 and S2). Lastly, most prey species reported in this study are species frequently to commonly found in reef-assemblages in Florida waters (Smith et al., 1975; Darcy & Gutherz, 1984; Matheson et al., 2017). These patterns thus give credence to the Harms-Tuohy, Schizas & Appeldoorn (2016) suggestion that lionfish diets are a result of regional ichtyofauna and their preferences for specific traits. As such, continued research is important to elucidate lionfish diets in unstudied regions and habitats to understand local impacts on species and communities.

Similarly, future studies should investigate whether lionfish of various size classes prey upon different taxa. Although ontogeny appears unlikely to have affected results reported herein given the size distribution of lionfish sampled, ontogenetic shifts in diet are known to occur in lionfish with studies indicating a shift from an invertebrate-to a fish dominated diet with increase in size (Morris & Akins, 2009; Muñoz, Currin & Whitfield, 2011; Dahl & Patterson, 2014; O’Farrell et al., 2014; Peake et al., 2018; Sancho et al., 2018; Malpica-Cruz, Green & Côté, 2019). For instance, Peake et al. (2018) reported significant negative and positive relationships between lionfish size (as measured by standard length) and the contributions of shrimp and fish prey respectively to lionfish diets in the Arrecifes de Cozumel National Park. Similarly, Sancho et al. (2018) reported that in Biscayne Bay National Park, lionfish sized <180 mm TL exhibited diets dominated by shrimp prey as measured by Index of Relative Importance and those larger than 180 mm TL exhibited diets dominated by fish. Sancho et al. (2018) also reported some differences in prey composition across size classes (e.g., pleocyemate shrimp only preyed by smaller lionfish, dendrobranchiate shrimp by larger classes); however, the extent to which ontogeny affects the composition of lionfish prey remains unclear. Future barcoding studies may aid in elucidating this point as well as the drivers of any such changes.

## Conclusion

In this study, we provide the first report of the lionfish prey-items from the Florida coastlines of the Gulf of Mexico. Our results are largely congruent with previous findings that suggest lionfish are generalists whose diet reflects regional species compositions and prey preferences for specific behavioral and morphological traits. Despite a limited number of samples and collection period, the results of our study were quite similar to the taxonomic composition of prey items reported by Dahl et al. (2017) from the Northern Gulf of Mexico, and suggest the former to be the most likely explanation. We report eleven putative novel prey species of lionfish, possibly reflecting the local prey availability in the habitat where lionfish were collected. We suggest molecular based efforts to understand the dietary habits of lionfish throughout their non-native region continue, with lionfish derbies serving as a cost-effective way to leverage public events into producing scientific information.

## Supplemental Information

10.7717/peerj.9922/supp-1 Supplemental Information 1 List of fish prey species identified from lionfish (*Pterois* spp.) stomachs from Western Atlantic habitats using barcoding approaches. Regions studied include: Western Florida (this study), the Yucatan Peninsula (Valdez-Moreno et al., 2012), The Bahamas (Côté et al., 2013), Belize (Rocha et al., 2015), Puerto Rico (Harms-Tuohy, Schizas & Appeldoorn, 2016), the Northern Gulf of Mexico (Dahl et al., 2012), and Byscaine National Park (Sancho et al., 2018). The prey list for this last study may be partial, as not all species identified were reported in the study.

10.7717/peerj.9922/supp-2 Supplemental Information 2 List of crustacean prey species identified from lionfish (*Pterois* spp.) stomachs from Western Atlantic habitats using barcoding approaches. Although barcoding efforts have been carried out in Western Florida (this study), the Yucatan Peninsula (Valdez-Moreno et al., 2012), The Bahamas (Côté et al., 2013), Belize (Rocha et al., 2015), Puerto Rico (Harms-Tuohy, Schizas & Appeldoorn, 2016), the Northern Gulf of Mexico (Dahl el al., 2012), and Byscaine National Park (Sancho et al., 2018), only three studies have reported identified crustacean species. The prey list for Sancho et al. (2018) may be partial, as not all species identified were reported in the study.

10.7717/peerj.9922/supp-3 Supplemental Information 3 All sequences obtained from lionfish prey items.

## Additional Information and Declarations

### Data Availability

The following information was supplied regarding data availability: Raw data is available as a Supplemental File.
