# Supplementary material for: First report of lionfish prey from Western Florida waters as identified by DNA barcoding
Source: PeerJ. 2020 Sep 11;8:e9922. doi: 10.7717/peerj.9922 (PMC7489240; doi:10.7717/peerj.9922)
Supplement: Supplemental Information 1 — Regions studied include: Western Florida (this study), the Yucatan Peninsula (Valdez-Moreno et al., 2012), The Bahamas (Côté et al., 2013), Belize (Rocha et al., 2015), Puerto Rico (Harms-Tuohy, Schizas & Appeldoorn, 2016), the Northern Gulf of Mexico (Dahl et al., 2012), and Byscaine National Park (Sancho et al., 2018). The prey list for this last study may be partial, as not all species identified were reported in the study. [file peerj-08-9922-s001.docx]

**Supplementary Table 1: List of fish prey species identified from lionfish (*Pterois* spp.) stomachs from Western Atlantic habitats using barcoding approaches.** Regions studied include: Western Florida (this study), the Yucatan Peninsula (Valdez-Moreno et al. 2012), The Bahamas (Côté et al. 2013), Belize (Rocha et al. 2015), Puerto Rico (Harms-Tuohy et al. 2016), the Northern Gulf of Mexico (Dahl el al. 2012), and Byscaine National Park (Sancho et al. 2018). The prey list for this last study may be partial, as not all species identified were reported in the study.

| **Family** | **Species** | **This**  **Study** | **Valdez-Moreno**  **et al. 2012** | **Côté et al.**  **2013** | **Rocha et al**  **2015** | **Harms-Tuohy et al.**  **2016** | **Dahl et al.**  **2017** | **Sancho et al.**  **2018** | **Number of Studies Reported In** |
| --- | --- | --- | --- | --- | --- | --- | --- | --- | --- |
| Acanthuridae | *Acanthurus tractus* |  |  |  |  | X |  |  | 1 |
| Antennariidae | *Fowlerichthys radiosus* |  |  |  |  |  | X |  | 1 |
| Apogonidae | *Apogon affinis* |  |  |  |  |  | X |  | 1 |
| Apogonidae | *Apogon aurolineatus* | X |  |  |  |  | X |  | 1 |
| Apogonidae | *Apogon binotatus* |  |  | X | X |  |  |  | 1 |
| Apogonidae | *Apogon lachneri* |  | X |  |  |  |  |  | 1 |
| Apogonidae | *Apogon maculatus* | X | X |  |  | X | X |  | 1 |
| Apogonidae | *Apogon mosavi* |  | X |  |  |  |  |  | 1 |
| Apogonidae | *Apogon phenaxa* |  |  | X |  |  |  |  | 1 |
| Apogonidae | *Apogon pillionatus* |  |  |  |  | X |  |  | 1 |
| Apogonidae | *Apogon pseudomaculatus* |  |  |  |  |  | X |  | 1 |
| Apogonidae | *Apogon sp.* |  |  |  |  |  | X |  | 1 |
| Apogonidae | *Apogon townsendi* |  | X | X |  | X |  |  | 1 |
| Apogonidae | *Astrapogon sp.* |  |  |  |  |  | X |  | 1 |
| Apogonidae | *Phaeoptyx conklini* |  |  |  |  | X |  |  | 1 |
| Apogonidae | *Phaeoptyx pigmentaria* |  |  | X |  | X | x |  | 1 |
| Apogonidae | *Phaeoptyx xenus* | X |  |  |  |  |  |  | 1 |
| Astrapogonidae | *Astrapogon puncticulatus* |  | X |  |  |  |  |  | 1 |
| Astrapogonidae | Unidentified species |  | X |  |  |  |  |  | 1 |
| Aulostomidae | *Aulostomus maculatus* |  |  | X |  |  |  |  | 1 |
| Blennidae | *Parablennius marmoreus* | X |  |  | X |  | X |  | 1 |
| Bothidae | *Bothus lunatus* |  | X |  |  |  |  |  | 1 |
| Bothidae | *Bothus ocellatusa* |  |  | X |  |  |  |  | 1 |
| Bothidae | *Bothus robinsi* |  |  |  |  |  | X |  | 1 |
| Callionymidae | *Diplogrammus pauciradiatus* | X |  |  |  |  |  |  | 1 |
| Carangidae | *Decapterus punctatus* | X |  |  |  |  | X |  | *1* |
| Carangidae | *Decapterus sp.* |  |  |  |  |  | X |  | *1* |
| Carangidae | *Trachurus lathami* |  |  |  |  |  | X |  | 1 |
| Chaenopsidae | *Acanthemblemaria asperaa* |  |  | X |  |  |  |  | 1 |
| Chaenopsidae | *Chaenopsis sp.* | X |  |  |  |  |  |  | 1 |
| Chaenopsidae | *Emblemariopsis arawak* |  |  |  |  | X |  |  | 1 |
| Chaenopsidae | *Emblemariopsis spp.* |  |  |  |  | X |  |  | 1 |
| Chaetodontidae | *Chaetodon capistratus* |  |  |  |  | X |  |  | 1 |
| Gobiidae | *Coryphopterus bol* |  |  | X |  |  |  |  | 1 |
| Gobiidae | *Coryphopterus eidolon* |  | X | X |  |  |  |  | 1 |
| Gobiidae | *Coryphopterus glaucofraenum* |  |  | X | X | X |  | X | 1 |
| Gobiidae | *Coryphopterus hyalinus* |  | X | X |  | X |  | X | 1 |
| Gobiidae | *Coryphopterus lipernes* |  |  |  |  | X |  | X | 1 |
| Gobiidae | *Coryphopterus personatus* |  |  | X | X | X |  |  | 1 |
| Gobiidae | *Coryphopterus sp.* | X |  |  |  |  |  |  | 1 |
| Gobiidae | *Coryphopterus thrix* |  | X |  |  |  |  |  | 1 |
| Gobiidae | *Coryphopterus tortugae* |  | X |  |  | X |  |  | 1 |
| Gobiidae | *Coryphopterus venezuelae* |  | X |  |  |  |  |  | 1 |
| Gobiidae | *Gnatholepsis thompsoni* |  |  | X |  | X |  |  | 1 |
| Gobiidae | *Lythrypnus minimus* |  | X |  |  |  |  |  | 1 |
| Gobiidae | *Lythrypnus spilus* |  |  | X |  |  |  |  | 1 |
| Gobiidae | *Microgobius carri* |  |  |  |  |  | X |  | 1 |
| Gobiidae | *Microgobius sp.* | X |  |  |  |  |  |  | 1 |
| Gobiidae | *Priolepis hipoliti* |  | X | X |  |  |  |  | 1 |
| Gobiidae | *Coryphopterus sp 2* |  |  |  |  |  | X |  | 1 |
| Grammatidae | *Gramma loreto* |  | X | X |  | X |  |  | 1 |
| Haemulidae | *Haemulon aurolineatum* | X |  |  |  |  | X |  | *1* |
| Haemulidae | *Haemulon flavolineatum* |  | X |  |  | X |  |  | *1* |
| Haemulidae | *Haemulon sciurus* |  |  |  |  |  |  | X | 1 |
| Holocentridae | *Holocentrus rufusa* |  |  | X |  |  |  |  | *1* |
| Holocentridae | *Sargocentron coruscum* |  | X | X |  | X |  |  | 1 |
| Inermiidae | *Inermia vittataa* |  |  | X |  |  |  |  | 1 |
| Labridae | *Bodianus rufus* |  |  |  |  | X |  |  | 1 |
| Labridae | *Clepticus parrae* |  |  | X |  | X |  |  | 1 |
| Labridae | *Halichoeres bathyphilus* |  |  |  |  |  | X |  | 1 |
| Labridae | *Halichoeres bivittatus* | X |  | X | X |  | X |  | 1 |
| Labridae | *Halichoeres garnoti* |  | X | X |  | X |  | X | 1 |
| Labridae | *Halichoeres maculipinna* |  |  | X |  |  |  |  | 1 |
| Labridae | *Halichoeres socialis* |  |  |  | X |  |  |  | 1 |
| Labridae | *Halichoeres sp. 2* |  |  |  |  |  | X |  | 1 |
| Labridae | *Halichoeres sp.* | X |  |  |  |  |  |  | 1 |
| Labridae | *Thalassoma bifasciatum* |  | X | X |  |  |  | X | 1 |
| Labridae | *Xyrichtys novacula* |  |  |  |  |  | X |  | 1 |
| Labrisomidae | *Labrisomus haitiensisa* |  |  | X |  |  |  |  | 1 |
| Labrisomidae | *Labrisomus sp.* |  |  |  | X |  |  |  | 1 |
| Labrisomidae | *Malacoctenus boehlkei* |  |  | X |  |  |  |  | 1 |
| Labrisomidae | *Malacoctenus macropus* |  |  |  |  | X |  |  | 1 |
| Labrisomidae | *Malacoctenus triangulates* |  | X |  |  |  |  |  | 1 |
| Labrisomidae | *Starksia langi* |  | X |  |  |  |  |  | 1 |
| Labrisomidae | *Starksia occidentalis* |  |  |  | X |  |  |  | 1 |
| Labrisomidae | *Starksia ocellata* |  | X |  |  |  |  |  | 1 |
| Labrisomidae | *Starksia williamsi* |  |  |  |  | X |  |  | 1 |
| Lutjanidae | *Lutjanidae sp.* |  |  |  |  | X |  |  | 1 |
| Lutjanidae | *Lutjanus campechanus* |  |  |  |  |  | X |  | 1 |
| Lutjanidae | *Lutjanus synagris* |  |  |  |  |  |  | X | 1 |
| Lutjanidae | *Ocyurus chrysurus* |  |  |  |  |  |  | X | 1 |
| Lutjanidae | *Pristipomoides aquilonaris* |  |  |  |  |  | X |  | 1 |
| Lutjanidae | *Rhomboplites aurorubens* |  |  |  |  |  | X |  | 1 |
| Microdesmidae | *Ptereleotris calliura* | X |  |  |  |  | X |  | 1 |
| Monacanthidae | *Monacanthus ciliatus* | X |  |  | X |  |  |  | 1 |
| Monacanthidae | *Monacanthus tuckeri* |  | X | X |  |  |  |  | 1 |
| Moncanthidae | *Monacanthus sp.* |  |  |  |  |  | X |  | 1 |
| Opistognathidae | *Opistognathus robinsi* |  |  |  |  |  | X |  | 1 |
| Paralichthyidae | *Cyclopsetta fimbriata* |  |  |  |  |  | X |  | 1 |
| Paralichthyidae | *Paralichthys albigutta* |  |  |  |  |  | X |  | 1 |
| Paralichthyidae | *Syacium papillosum* |  |  |  |  |  | X |  | 1 |
| Paralichthyidae | *Syacium sp.* |  |  |  |  |  | X |  | 1 |
| Pomacentridae | *Abudefduf saxatilis* |  | X |  |  |  |  |  | 1 |
| Pomacentridae | *Chromis cyanea* |  |  | X |  | X |  |  | 1 |
| Pomacentridae | *Chromis enchrysurus* |  |  |  |  |  | X |  | 1 |
| Pomacentridae | *Chromis multilineata* |  |  | X |  | X |  |  | 1 |
| Pomacentridae | *Chromis scotti* | X |  |  |  |  | X |  | 1 |
| Pomacentridae | *Chromis sp.* |  |  |  |  |  | X |  | 2 |
| Pomacentridae | *Stegastes fuscus* |  |  |  |  |  | X |  | 2 |
| Pomacentridae | *Stegastes partitus* |  | X | X |  | X |  | X | 2 |
| Pomacentridae | *Stegastes variabilis* | X |  | X | X | X | X |  | *2* |
| Priacanthidae | *Heteropriacanthus cruentatus* |  |  |  |  | X |  |  | 2 |
| Scaridae | *Scarus iseri* |  | X |  | X |  |  |  | 2 |
| Scaridae | *Sparisoma atomarium* | X |  |  | X |  |  |  | 2 |
| Scaridae | *Sparisoma aurofrenatum* |  | X | X | X |  |  |  | 2 |
| Scaridae | *Sparisoma radians* |  |  |  |  | X |  |  | 2 |
| Scaridae | *Sparisoma viride* |  | X |  | X | X |  |  | 2 |
| Scaridea | *Scarus iseri* |  |  |  |  | X |  |  | 2 |
| Scaridea | *Scarus taeniopterus* |  | X |  |  | X |  |  | 2 |
| Scaridea | *Scarus vetula* |  |  |  |  | X |  |  | 2 |
| Scorpaenidae | *Scorpaena brasiliensis* |  |  |  |  |  | X |  | 2 |
| Serranidae | *Centropristis ocyurus* |  |  |  |  |  | X |  | 2 |
| Serranidae | *Centropristis sp.* |  |  |  |  |  | X |  | 2 |
| Serranidae | *Cephalopholis cruentata* |  | X | X |  |  |  |  | 2 |
| Serranidae | *Diplectrum formosum* | X |  |  |  |  | X |  | 2 |
| Serranidae | *Diplectrum sp.* |  |  |  |  |  | X |  | 2 |
| Serranidae | *Hypoplectrus aberrans* |  |  |  |  | X |  |  | 2 |
| Serranidae | *Hypoplectrus floridae* | X |  |  |  |  |  |  | 2 |
| Serranidae | *Hypoplectrus nigricans* |  |  |  |  | X |  |  | 2 |
| Serranidae | *Hypoplectrus puella* |  |  |  | X | X |  |  | 2 |
| Serranidae | *Hypoplectrus spp.* |  |  |  |  | X |  |  | 2 |
| Serranidae | *Liopropoma rubre* |  | X | X |  |  |  |  | 2 |
| Serranidae | *Pronotogrammus martinicensis* |  |  |  |  |  | X |  | 2 |
| Serranidae | *Serraniculus sp.* |  |  |  |  |  | X |  | 3 |
| Serranidae | *Serranus flaviventris* |  |  |  | X |  |  |  | 3 |
| Serranidae | *Serranus subligarius* |  |  |  |  |  | X |  | 3 |
| Serranidae | *Serranus tigrinus* |  |  | X |  |  |  |  | 3 |
| Sparidae | *Pagrus pagrus* |  |  |  |  |  | X |  | 3 |
| Synodontidae | *Saurida brasiliensis* |  |  |  |  |  | X |  | 3 |
| Synodontidae | *Synodus intermedius* | X |  |  |  | X |  |  | 3 |
| Synodontidae | *Synodus macrostigmus* |  |  |  |  |  | X |  | 3 |
| Synodontidae | *Synodus poeyi* |  |  |  |  |  | X |  | 3 |
| Synodontidae | *Synodus saurus* | X |  |  |  |  |  |  | 4 |
| Synodontidae | *Synodus synodus* |  |  | X |  |  | X |  | 4 |
| Triglidae | *Bellator brachychir* |  |  |  |  |  | X |  | 4 |
| Triglidae | *Bellator militaris* |  |  |  |  |  | X |  | 4 |
| Triglidae | *Prionotus sp.* |  |  |  |  |  | X |  | 4 |
| Tripterygiidae | *Enneanectes altivelis* |  | X |  |  |  |  |  | 4 |
| Tripterygiidae | *Enneanectes boehlkei* |  | X |  |  |  |  |  | 5 |
