# Supplementary material for: First report of lionfish prey from Western Florida waters as identified by DNA barcoding
Source: PeerJ. 2020 Sep 11;8:e9922. doi: 10.7717/peerj.9922 (PMC7489240; doi:10.7717/peerj.9922)
Supplement: Supplemental Information 2 — Although barcoding efforts have been carried out in Western Florida (this study), the Yucatan Peninsula (Valdez-Moreno et al., 2012), The Bahamas (Côté et al., 2013), Belize (Rocha et al., 2015), Puerto Rico (Harms-Tuohy, Schizas & Appeldoorn, 2016), the Northern Gulf of Mexico (Dahl el al., 2012), and Byscaine National Park (Sancho et al., 2018), only three studies have reported identified crustacean species. The prey list for Sancho et al. (2018) may be partial, as not all species identified were reported in the study. [file peerj-08-9922-s002.docx]

**Supplementary Table 2: List of crustacean prey species identified from lionfish (*Pterois* spp.) stomachs from Western Atlantic habitats using barcoding approaches.** Although barcoding efforts have been carried out in Western Florida (this study), the Yucatan Peninsula (Valdez-Moreno et al. 2012), The Bahamas (Côté et al. 2013), Belize (Rocha et al. 2015), Puerto Rico (Harms-Tuohy et al. 2016), the Northern Gulf of Mexico (Dahl el al. 2012), and Byscaine National Park (Sancho et al. 2018), only three studies have reported identified crustacean species. The prey list for Sancho et al. (2018) may be partial, as not all species identified were reported in the study.

| **Family** | **Species** | **This Study** | **Valdez-Moreno et al.**  **2012** | **Sancho et al.**  **2018** | **Number of Studies Reported In** |
| --- | --- | --- | --- | --- | --- |
| Alpheidae | *Synalpheus* sp. |  | X |  | 1 |
| Alpheidae | Unidentified species | X |  |  | 1 |
| Euphasiidae | *Euphasia* sp. |  | X |  | 1 |
| Gammaridae | Unidentified species | X |  |  | 1 |
| Gonodactylidae | *Neogonodactylus bredini* | X |  |  | 1 |
| Gonodactylidae | Unidentified species |  | X |  | 1 |
| Hyppolytidae | *Thor* sp. |  | X |  | 1 |
| Menippidae | *Menippe mercenaria* |  |  | X | 1 |
| Palaemonidae | Unidentified species | X | X |  | 2 |
| Penaidae | *Farfantepenaeus duorarum* |  |  | X | 1 |
| Penaidae | *Metapenaeopsis goodei* | X |  |  | 1 |
| Portunidae | *Achelous ordwayi* | X |  |  | 1 |
| Pseudosquillidae | *Pseudosquilla* sp. |  | X |  | 1 |
